# β-caryophyllene attenuates lead acetate-induced hepatotoxicity through regulation of oxidative stress, inflammation, and PI3K/AKT/mTOR signaling

**DOI:** 10.1007/s00210-026-05605-1

**Published:** 2026-06-25

**Authors:** Esra Manavoğlu Kirman, İsmail Bolat, Tuba Karaarslan, Samet Tekin, Aslıhan Atasever

**Affiliations:** 1 Department of Pathology, Faculty of Veterinary Medicine, Atatürk University, Erzurum, Türkiye; 2 Department of Biochemistry, Faculty of Veterinary Medicine, Atatürk University, Erzurum, Türkiye; 3 Department of Physiology, Faculty of Veterinary Medicine, Atatürk University, Erzurum, Türkiye; 4 Department of Veterinary Medicine, Çayırlı Vocational High School, Erzincan Binali Yıldırım University, Erzincan, Türkiye

**Keywords:** β-Caryophyllene, Lead acetate, Hepatotoxicity, Oxidative stress, PI3K/AKT/mTOR signaling, Apoptosis

## Abstract

Lead acetate (PbAc) is an environmental toxicant known to induce liver injury through oxidative stress, inflammation, and apoptosis. This study aimed to investigate the potential hepatoprotective effects of β-caryophyllene (BCP), a natural sesquiterpene with antioxidant and anti-inflammatory properties, against PbAc-induced hepatotoxicity and to explore the underlying molecular mechanisms. Sixty male Sprague–Dawley rats were randomly assigned to five groups: control, BCP200, PbAc, PbAc + BCP100, and PbAc + BCP200. PbAc was used to induce hepatotoxicity, while BCP was administered at doses of 100 and 200 mg/kg. Serum liver enzymes (ALT, AST, ALP, and GGT), oxidative stress parameters (MDA, SOD, and GSH), and inflammatory cytokines (TNF-α, IL-1β, and IL-6) were evaluated. Immunohistochemical and immunofluorescence analyses were performed to assess Nrf2, HO-1, Sirt1, Keap1, and 8-OHdG expression. In addition, the mRNA expression of PI3K, AKT, mTOR, Bax, Bcl-2, and Caspase-3 was determined by qRT-PCR, and histopathological examinations of liver tissue were conducted. PbAc exposure significantly increased serum liver enzyme levels, lipid peroxidation, and pro-inflammatory cytokines while reducing antioxidant defense markers. It also suppressed Nrf2/HO-1 signaling and PI3K/AKT/mTOR gene expression, accompanied by increased Bax and Caspase-3 and decreased Bcl-2 expression, indicating enhanced apoptosis. Histopathological analysis confirmed severe hepatic degeneration and necrosis. BCP treatment significantly attenuated these alterations by reducing oxidative stress and inflammatory responses, restoring Nrf2/HO-1 signaling, improving PI3K/AKT/mTOR pathway activity, and regulating apoptosis-related gene expression. These findings suggest that β-caryophyllene may exert protective effects against PbAc-induced hepatotoxicity through modulation of oxidative stress, inflammation, cell survival signaling, and apoptosis pathways, highlighting its potential as a supportive therapeutic candidate for heavy metal-induced liver injury.

**Graphical Abstract:**

**Figure:**
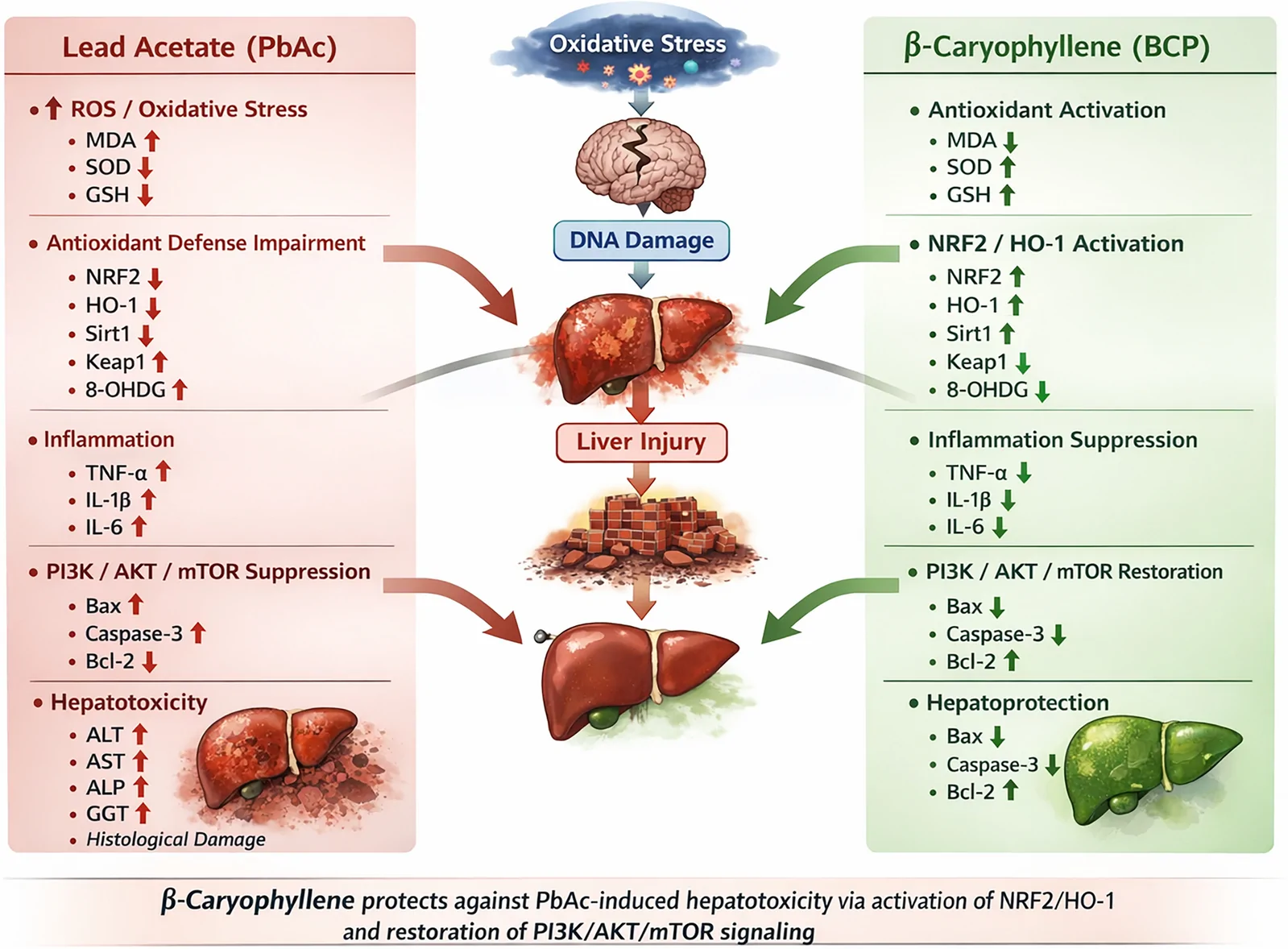

## Introduction

Lead (Pb) is one of the toxic heavy metals commonly found in the environment and can accumulate in living organisms because it is not biodegradable. Humans and animals are primarily exposed to lead through the consumption of contaminated food and water and through inhalation of lead-containing dust particles. The World Health Organization (WHO) reports that lead exposure is a significant public health issue and, according to 2016 data, more than 540,000 deaths worldwide are related to lead toxicity (Ileriturk et al. 2021). After being absorbed into the body, lead has been reported to accumulate in many tissues such as the liver, kidneys, nervous system, lungs, bones, gastrointestinal system, reproductive system, and cardiovascular system, causing various toxic effects in these tissues (Zheng et al. 2019; Luo et al. 2019; Balachandar et al. 2020; Bolat et al. 2025d).

The liver is a key target tissue for heavy metal toxicity because it plays a fundamental role in the metabolism and detoxification of xenobiotics. Lead acetate (PbAc), one of the commonly used forms of lead, has been reported to cause significant oxidative stress in hepatocytes and to increase liver enzymes such as serum alanine aminotransferase (ALT), aspartate aminotransferase (AST), alkaline phosphatase (ALP), and gamma-glutamyl transferase (GGT) (Patrick 2006; Flora et al. 2012). PbAc exposure can lead to excessive production of reactive oxygen species, resulting in lipid peroxidation, suppression of the antioxidant defense system, and DNA damage. In this process, an increase in malondialdehyde (MDA) levels is considered an important indicator of lipid peroxidation, while a decrease in superoxide dismutase (SOD) and glutathione (GSH) levels indicates a weakened antioxidant defense. Furthermore, the accumulation of 8-hydroxy-2'-deoxyguanosine (8-OHdG) is considered an important biomarker of oxidative DNA damage associated with lead exposure (Flora et al. 2012; Tekin et al. 2025).

It has been reported that the Keap1/Nrf2/HO-1 signaling pathway, one of the key regulators of cellular antioxidant defense, plays an important role in the development of lead-induced oxidative damage. Under oxidative stress conditions, the activation of Nrf2 enhances cellular defense by increasing the expression of cytoprotective genes such as HO-1. However, it has been shown that lead exposure also triggers an inflammatory response, increasing the production of proinflammatory cytokines such as IL-1β, TNF-α, and IL-6 (Gillis et al. 2012; Harshitha et al. 2024). Furthermore, it is thought that the PI3K/AKT/mTOR signaling pathway, which is critical for cell survival and metabolic regulation, may be disrupted during lead-induced cellular stress, contributing to the progression of damage in hepatocytes. Lead exposure may also activate apoptotic mechanisms by disrupting the Bax/Bcl-2 balance and leading to programmed cell death through the activation of Caspase-3, one of the executive caspases (Li et al. 2023; Bolat et al. 2025d).

In recent years, the protective effects of natural compounds against organ damage caused by heavy metals have been increasingly studied. One such compound, β-caryophyllene (BCP), is a natural sesquiterpene found in the essential oils of various plants and is known as a selective CB2 receptor agonist (Gertsch et al. 2008; Tekin et al. 2026). BCP has been reported to possess potent antioxidant and anti-inflammatory properties, potentially contributing to the reduction of oxidative stress by activating the Nrf2/HO-1 pathway (Mohamed et al. 2022; Bolat et al. 2025e).

This study aimed to investigate the potential hepatoprotective effects of β-caryophyllene in an experimental hepatotoxicity model induced by lead acetate. In this context, the effects of β-caryophyllene on lead-induced liver damage were evaluated through molecular mechanisms related to oxidative stress, antioxidant defense, inflammation, apoptotic processes, and the PI3K/AKT/mTOR signaling pathway.

## Materials and methods

### Chemicals

BCP (CAS No. 87–44–5) and PbAc (Cas No: 6080–56-4) were purchased from Sigma-Aldrich, USA. The ELISA kits employed in the experiments were supplied by YL-Biont (Shanghai, China) and included the Malondialdehyde (MDA) (YLA0029RA), Superoxide Dismutase (SOD) ELISA Kit (YLA0115RA), Glutathione (GSH) (YLA1511RA), Interleukin-1β (IL-1β) (YLA0030RA), Interleukin-6 (IL-6) (YLA0031RA), Rat and Rat Tumor Necrosis Factor-α (TNF-α) (YLA0118RA).

### Animals and experimental groups

All experimental procedures involving animals were approved by the Local Animal Experimentation Ethics Committee of Atatürk University (Approval No. 2025/14) and were conducted in accordance with national and international guidelines for the care and use of laboratory animals. The experimental design, conduct, and reporting of this animal study followed the ARRIVE 2.0 guidelines (Animal Research: Reporting of In Vivo Experiments) to enhance transparency, reproducibility, and methodological rigor in in vivo research. The experimental animals used in our study were obtained from Atatürk University Medical Experimental Research Center (ATADEM), and a total of 50 healthy, 12-week-old male Sprague Dawley rats weighing 220–290 g were used for this purpose. During the 7-day acclimatization period, all animals were housed under standard laboratory conditions with a controlled temperature (22 ± 2 °C), relative humidity (50–60%), and a 12 h light/12 h dark cycle. Rats had free access to standard pellet diet and water ad libitum. Animals were monitored daily to minimize stress and ensure their health status prior to the experimental procedures. After a 7-day adaptation period, the rats were randomly assigned to 5 experimental groups. Group 1 (Control group): Physiological saline was administered orally for 7 days. Group 2 (BCP, 200 mg/kg): BCP solution was administered intragastrically (IG) at the dose specified in the protocol for 7 days. The selected dose (200 mg/kg) was based on previous studies reporting its effective antioxidant and protective properties. This group was included to evaluate the potential effects of β-caryophyllene under normal physiological conditions and to confirm the absence of any adverse effects at this dose (Espinosa-Ahedo et al. 2022; Bolat et al. 2025a, b, c, d, e, f, g). Group 3 (PbAc, 30 mg/kg): Lead acetate (30 mg/kg) was administered as a single oral dose on the final day of the experimental protocol following a 24-h fasting period to induce acute hepatotoxicity. This experimental model was established based on previously published studies demonstrating that single-dose PbAc administration induces acute oxidative stress–mediated liver injury (Ileriturk et al. 2021). Group 4 (BCP 100 mg/kg + PbAc, 30 mg/kg): Rats were administered BCP (100 mg/kg) intragastrically for 7 days, followed by a single oral dose of PbAc (30 mg/kg) after 24-h fasting. Group 5 (BCP 200 mg/kg + PbAc, 30 mg/kg): Rats were administered BCP (200 mg/kg) intragastrically for 7 days, followed by a single oral dose of PbAc (30 mg/kg) after 24-h fasting.

Rats in all groups were sacrificed under general anesthesia 24 h after the last application, and liver tissues and blood sera were collected. Liver tissue was fixed in 10% neutral formalin for histopathological and immunohistochemical analyses. The collected liver tissues were stored at −80 °C for biochemical and molecular analyses.

### Determination of serum enzyms levels

Blood was centrifuged at 3500–4000 rpm for 10 min at 4 °C, and the serum was separated. The serum ALP, ALT, AST, and GGT enzyme activities were measured with commercial kits (TML, Diagnostic Medical Products).

### Homogenization of liver tissues

Liver tissue 1500 µl of PBS solution was added and homogenization. Samples were centrifuged at 5000 rpm for 10 min. The obtained supernatants transferred to Eppendorf.

### Biochemical analyses

#### Oxidative parameters and antioxidant enzymes analysis

Oxidative parameters and antioxidant enzyme activities were evaluated using an ELISA plate reader (Bio-Tek, Winooski, VT, USA) that can measure absorbance at a wavelength of 450 nm. Supernatants were analyzed according to the instructions provided by the respective ELISA kits to determine MDA, GSH and SOD.

#### Inflammation markers analysis

The levels of IL-1β, TNF-α, and IL-6 in the supernatants were measured as the protocols respective ELISA kits.

#### RT-PCR analysis

Total RNA was isolated from approximately 20 mg of tissue using 1,000 µL of QIAzol reagent (Qiagen, Germany). Tissue samples were first homogenized and then centrifuged at 12,000 rpm for 30 min, after which RNA extraction was completed following the manufacturer’s instructions. For complementary DNA (cDNA) synthesis, 2,000 ng of total RNA was reverse transcribed using the High-Capacity cDNA Reverse Transcription Kit according to the protocol provided by the manufacturer. Quantitative real-time PCR (qRT-PCR) analyses were carried out using Qiagen SYBR Green PCR Master Mix together with gene-specific primers targeting BAX, PI3K, Bcl-2, AKT, Caspase-3, and mTOR. GAPDH served as the internal reference gene for normalization. Relative gene expression levels were determined using the 2^ − ΔΔCt method (Bolat et al. 2025c). All qRT-PCR reactions were performed in triplicate to ensure reproducibility. The sequences of the primers used in the amplification reactions are listed in Table 1.

**Table 1:** Primer sequences used for real-time PCR

Gene	Sequence (5′ − 3′)	Accession number (NCBI)
Bax	F: GAGACACCTGAGCTGACCTT R: CGTCTGCAAACATGTCAGCT	NM_017059.2
Bcl-2	F: CCTGGCATCTTCTCCTTCCA R: GGACATCTCTGCAAAGTCGC	NM_016993.1
Caspase-3	F: CTTCATCATTCAGGCCTGCC R: CACGAGTGAGGATGTGCATG	NM_012922.2
PI3K	F: GCTTGTTCTGTGGTTGCAGA R: TAGTGGAGCACCAGCTCCTT	NM_022213
AKT	F: GAGTACTTGCACTCGACGGA R: CCATGAGGATGAGCTCGAAG	NM_017093.1
mTOR	F: AGGAAGGACGTTTGCTCAGA R: TCCCTCACTGAACACAGCAG	NM_019906
GAPDH	F: AGTCTACTGGCGTCTTCACC R: CCACGATGCCAAAGTTGTCA	NM_017008.4

### Histopathological examination

After the liver tissues were fixed in formalin solution, they were embedded in paraffin, and sections were taken. The sections were then stained with Hematoxylin and Eosin and the tissues were examined histopathologically. The examinations identified degenerative and necrotic changes that may occur in liver tissues. Following the determinations, the findings were scored using a scoring system: (0–4): 0 (0%) No lesion, 1 (1–10%) Very mild, 2 (11–25%) Mild, 3 (26–50%) Moderate, 4 (51–100%) Severe (Bolat et al. 2025a, b, c, d, e, f, g).

### Immunohistochemical examination

The initial steps in immunohistochemical staining were performed according to the procedure described by (Bolat et al. 2025a). Primary antibodies (Sirt1 Cat No: DF6033, Dilution Ratio: 1/100; Keap1 Cat No:  STJ96583, Dilution Ratio: 1/100; 8-OHdG Cat No: bs-1278R, Dilution Ratio: 1/200) were added dropwise and incubated according to the instructions for use. Following the application of secondary antibodies, 3-Amino-9-Ethylcarbazole (AEC) chromogen was added dropwise to the tissues. The stained sections were examined using a light microscope (Leica, Flexacam, i5).

### Immunofloresence exemination

The initial steps in immunofloresence staining were performed according to the procedure described by (Bolat et al. 2025b). Primary antibodies (Nrf2 Cat No: bs-1074R, Rabbit polyclonal, Dilution Ratio: 1/200; HO-1 Cat No: bs-2075R, Rabbit polyclonal, Dilution Ratio: 1/100) were added to the tissues and incubated according to the instructions for use. A secondary antibody was used as a secondary marker (Fluorescein isothiocyanate (FITC) Cat No: ab6717, Rabbit polyclonal, Dilution Ratio: 1/1000) and incubated in the dark for 45 min. Then, 4',6-diamidino-2-phenylindole (DAPI) (Cat No: ab104140) with mounting medium was added to the sections, incubated in the dark for 5 min, and the sections were covered with a coverslip. The stained tissues were examined under a fluorescence-equipped microscope (Zeiss AXIO, Germany). Positivity in immunohistochemical (IHC) and immunofluorescent (IF) staining was quantitatively assessed using the ImageJ analysis program. The staining intensity in the regions of interest (ROI) selected in the images obtained with the same microscope and camera settings for all groups was calculated based on the mean intensity value. The obtained mean values were used in statistical analyses. To enable reliable relative comparisons between groups, all images were recorded under standard and fixed imaging settings.

### Statistical analysis

First, the Shapiro–Wilk test was performed to determine the normal distribution of the results. The nonparametric Kruskal–Wallis test and Dunn's test were used to detect group interaction in H&E staining results. For parametric data, One-Way ANOVA followed by Tukey's test was performed. GraphPad Prism software was used for statistical analysis, and data were evaluated with p < 0.05 considered statistically significant.

## Results

### Effects of β-caryophyllene on serum liver enzyme levels

**Fig. 1:**
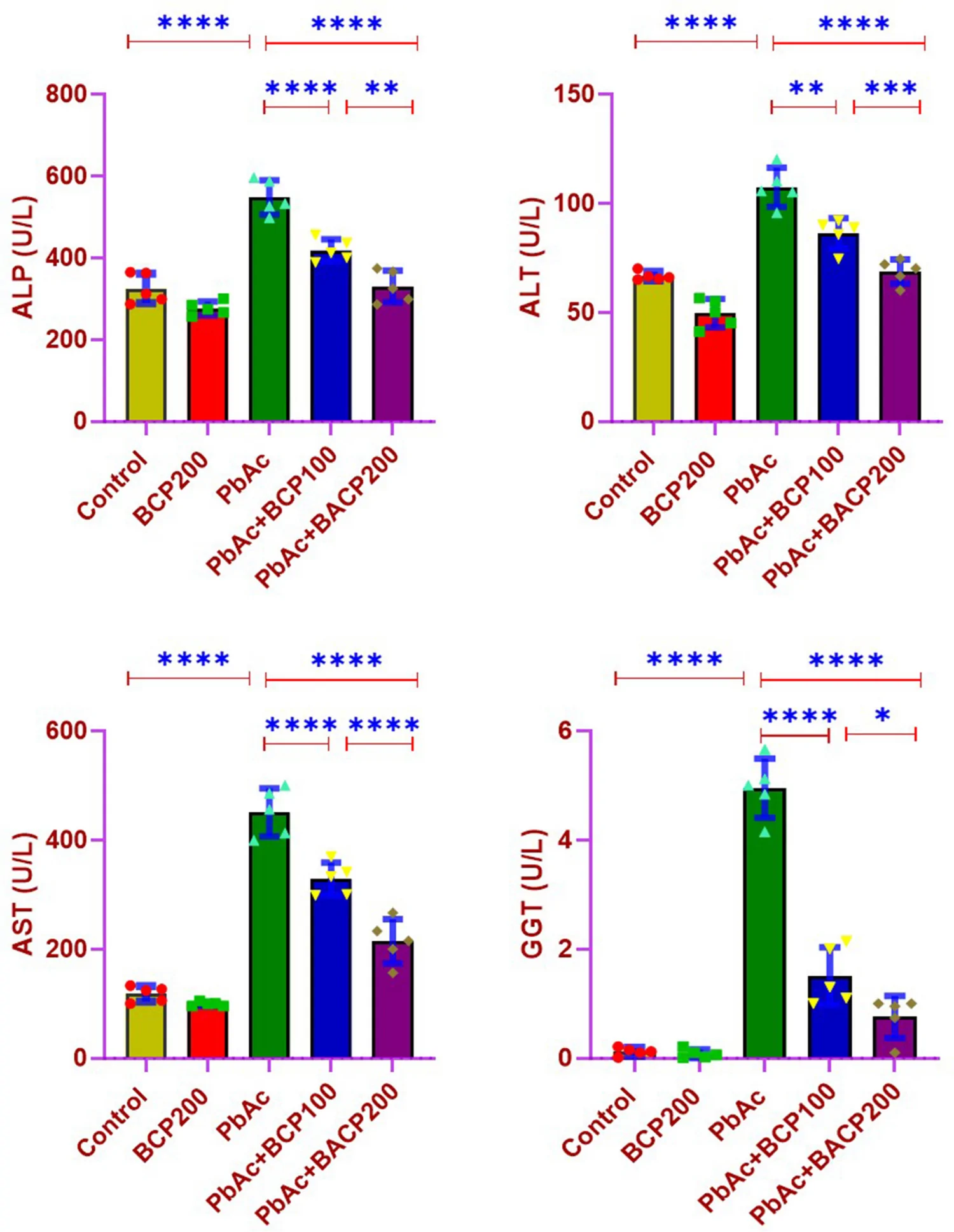
Effects of β-caryophyllene on serum liver enzyme levels in PbAc-induced hepatotoxicity. Serum levels of ALT, AST, ALP, and GGT in control, BCP200, PbAc, PbAc + BCP100, and PbAc + BCP200 groups. Data are presented as mean ± SD (n = 5). Statistical significance was determined using one-way ANOVA followed by Tukey’s post hoc test. *p < 0.05, **p < 0.01, ***p < 0.001, ****p < 0.0001; ns: not significant

Serum biochemical analyses revealed significant alterations in liver enzyme levels among the experimental groups (Fig. 1). The PbAc-treated group exhibited a marked increase in serum ALT, AST, ALP, and GGT levels compared with the control group (p < 0.05). Administration of β-caryophyllene attenuated these elevations in a dose-dependent manner. In particular, both PbAc + BCP100 and PbAc + BCP200 groups showed significantly lower ALT, AST, ALP, and GGT levels compared with the PbAc group (p < 0.05). Moreover, enzyme levels in the PbAc + BCP200 group were closer to those of the control group, indicating a stronger protective effect at the higher dose. No significant differences were observed between the control and BCP200 groups for any of the evaluated parameters (p > 0.05).

### Effects of β-caryophyllene on oxidative stress and antioxidant defense markers in PbAc-induced hepatotoxicity

**Fig. 2:**
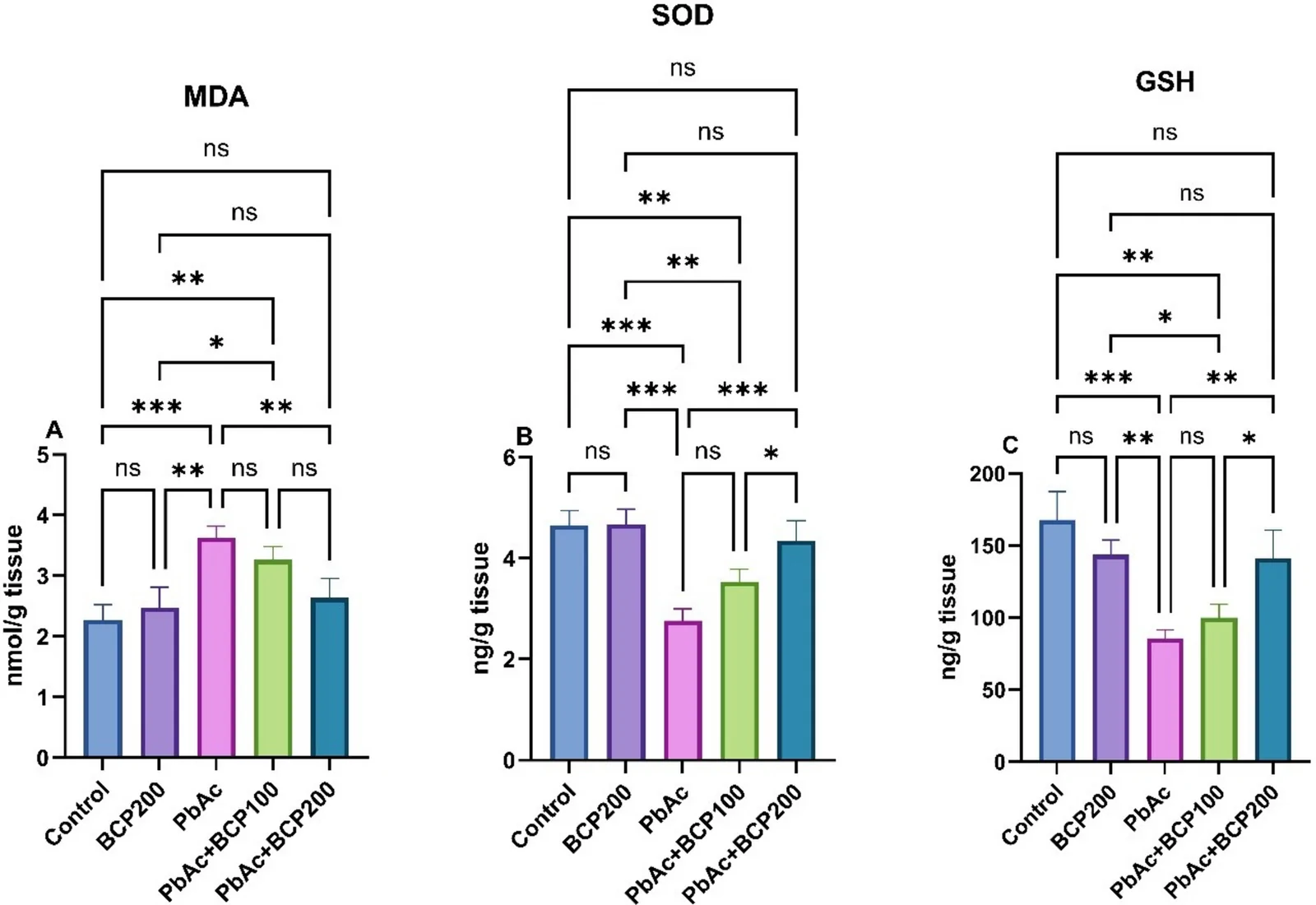
Effects of β-caryophyllene on oxidative stress parameters. Levels of MDA, SOD, and GSH in liver tissue of experimental groups. Data are expressed as mean ± SD (n = 7). Statistical analysis was performed using one-way ANOVA followed by Tukey’s post hoc test. *p < 0.05, **p < 0.01, ***p < 0.001

Oxidative stress parameters were significantly altered in the PbAc-treated group compared with the control group (Fig. 2). PbAc administration significantly increased MDA levels, while markedly decreasing the antioxidant parameters SOD and GSH (p < 0.05). Treatment with β-caryophyllene significantly attenuated these alterations. In particular, the PbAc + BCP200 group showed a significant reduction in MDA levels and a marked increase in SOD and GSH levels compared with the PbAc group (p < 0.05).

**Fig. 3:**
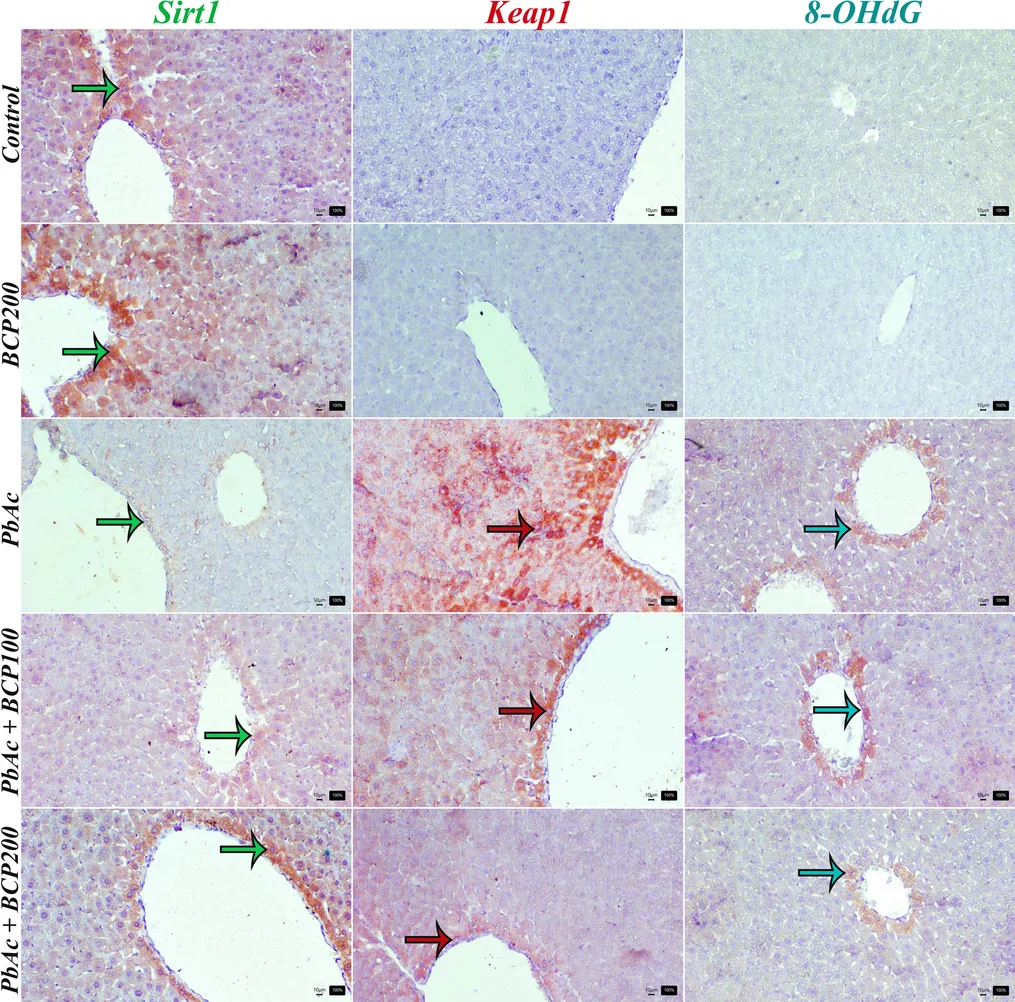
Immunohistochemical analysis of oxidative stress markers in liver tissue. Representative immunohistochemical staining of Sirt1, Keap1, and 8-OHdG in liver tissues from control, BCP200, PbAc, PbAc + BCP100, and PbAc + BCP200 groups. Sirt1 (green arrow) expression while increasing Keap1 (red arrow) and 8-OHdG (turquoise arrow) immunoreactivity. Images were obtained at × 20 magnification. Scale bar: 10 µm

Immunohistochemical findings further supported these results. The PbAc group exhibited reduced Sirt1 immunoreactivity together with markedly increased Keap1 and 8-OHdG expression compared with the control group (Fig. 3). In contrast, BCP treatment significantly increased Sirt1 expression and reduced Keap1 and 8-OHdG immunoreactivity in the PbAc-treated groups.

**Fig. 4:**
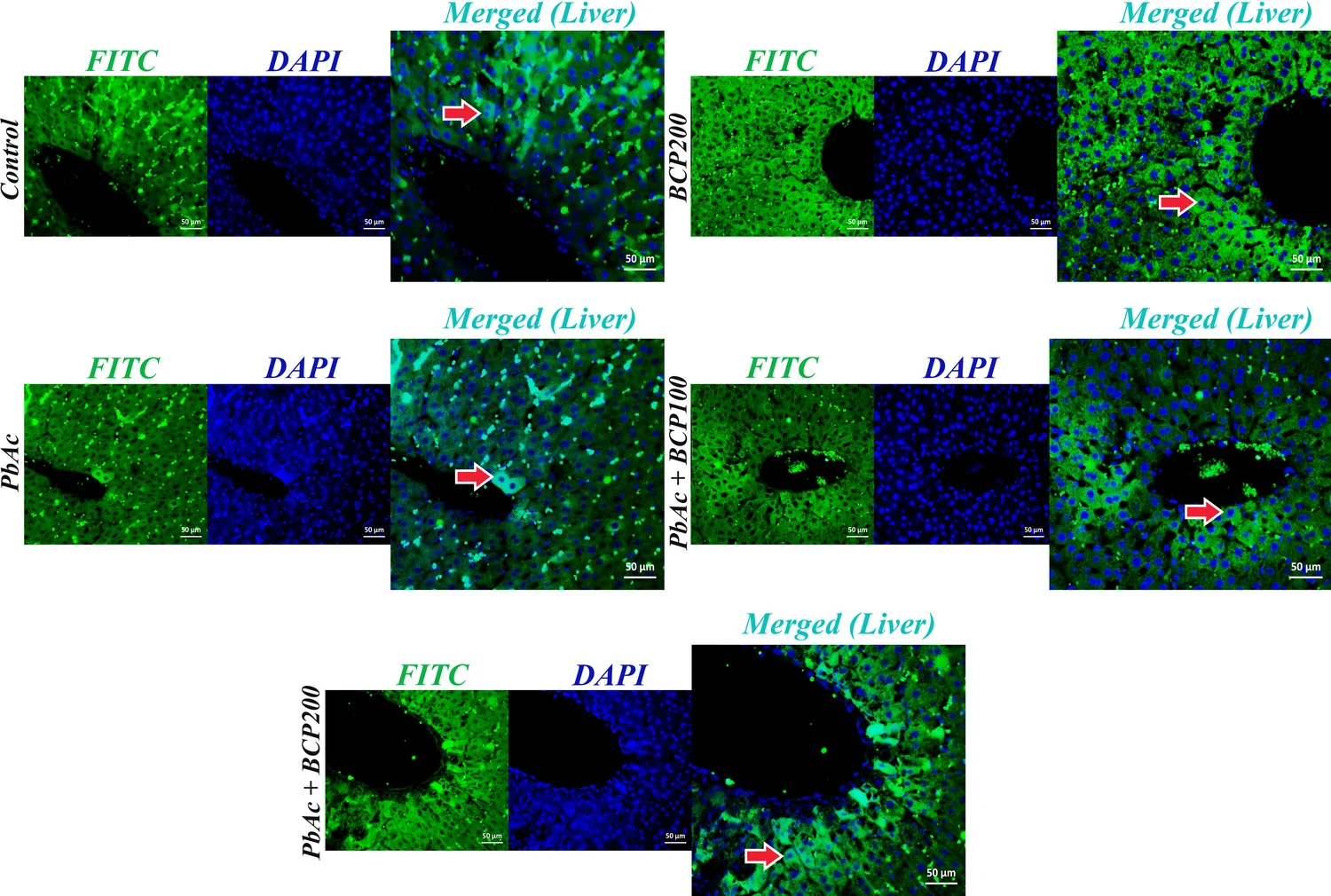
Immunofluorescence analysis of Nrf2 expression in liver tissue. Representative immunofluorescence images of Nrf2 (red arrow) expression in liver tissue sections. Green fluorescence indicates target protein expression (FITC), while nuclei are counterstained with DAPI (blue). Scale bar: 50 μm

**Fig. 5:**
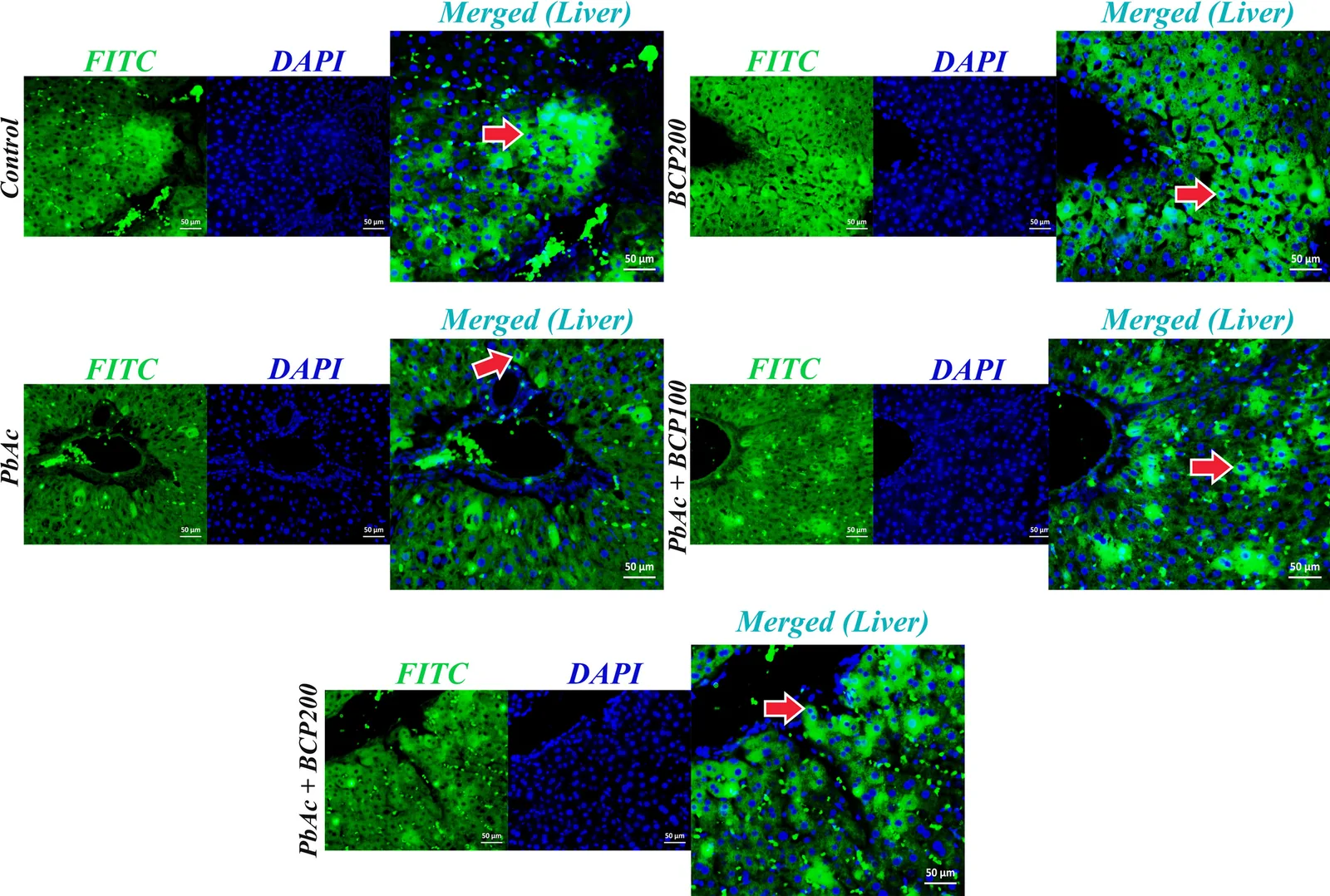
Immunofluorescence analysis of HO-1 expression in liver tissue. Representative immunofluorescence images of HO-1 (red arrow) expression in liver tissue sections. Green fluorescence indicates target protein expression (FITC), while nuclei are counterstained with DAPI (blue). Scale bar: 50 μm

Consistent with these findings, immunofluorescence analysis demonstrated that Nrf2 and HO-1 fluorescence intensities were markedly decreased in the PbAc group, whereas BCP treatment significantly increased Nrf2 and HO-1 expression levels (Figs. 4 and 5). These changes were more pronounced in the PbAc + BCP200 group.

**Fig. 6:**
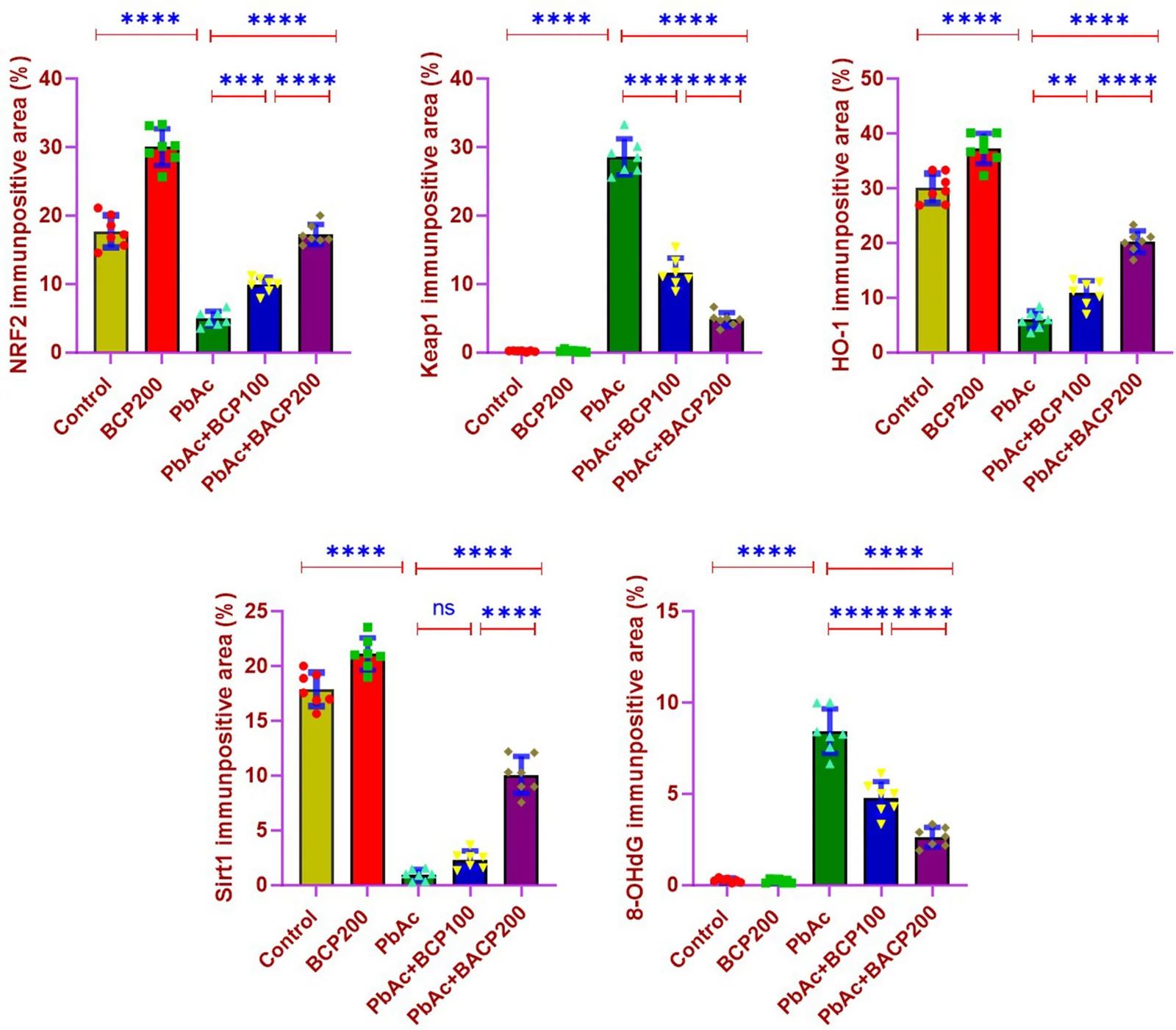
Quantitative analysis of immunopositive areas. Quantification of Nrf2, HO-1, Keap1, Sirt1, and 8-OHdG immunopositive areas (%) in liver tissues of experimental groups. Data are presented as mean ± SD (n = 7). Statistical analysis was performed using one-way ANOVA followed by Tukey’s post hoc test.*p < 0.05, **p < 0.01, ***p < 0.001, ****p < 0.0001

Quantitative analysis of immunopositive areas confirmed these observations. Nrf2, HO-1 and Sirt1 levels were significantly decreased in the PbAc group, while Keap1 and 8-OHdG levels were significantly increased compared with the control group. Administration of β-caryophyllene significantly reversed these alterations, particularly in the PbAc + BCP200 group (Fig. 6).

### Effects of β-caryophyllene on inflammatory cytokines in PbAc-induced hepatotoxicity

**Fig. 7:**
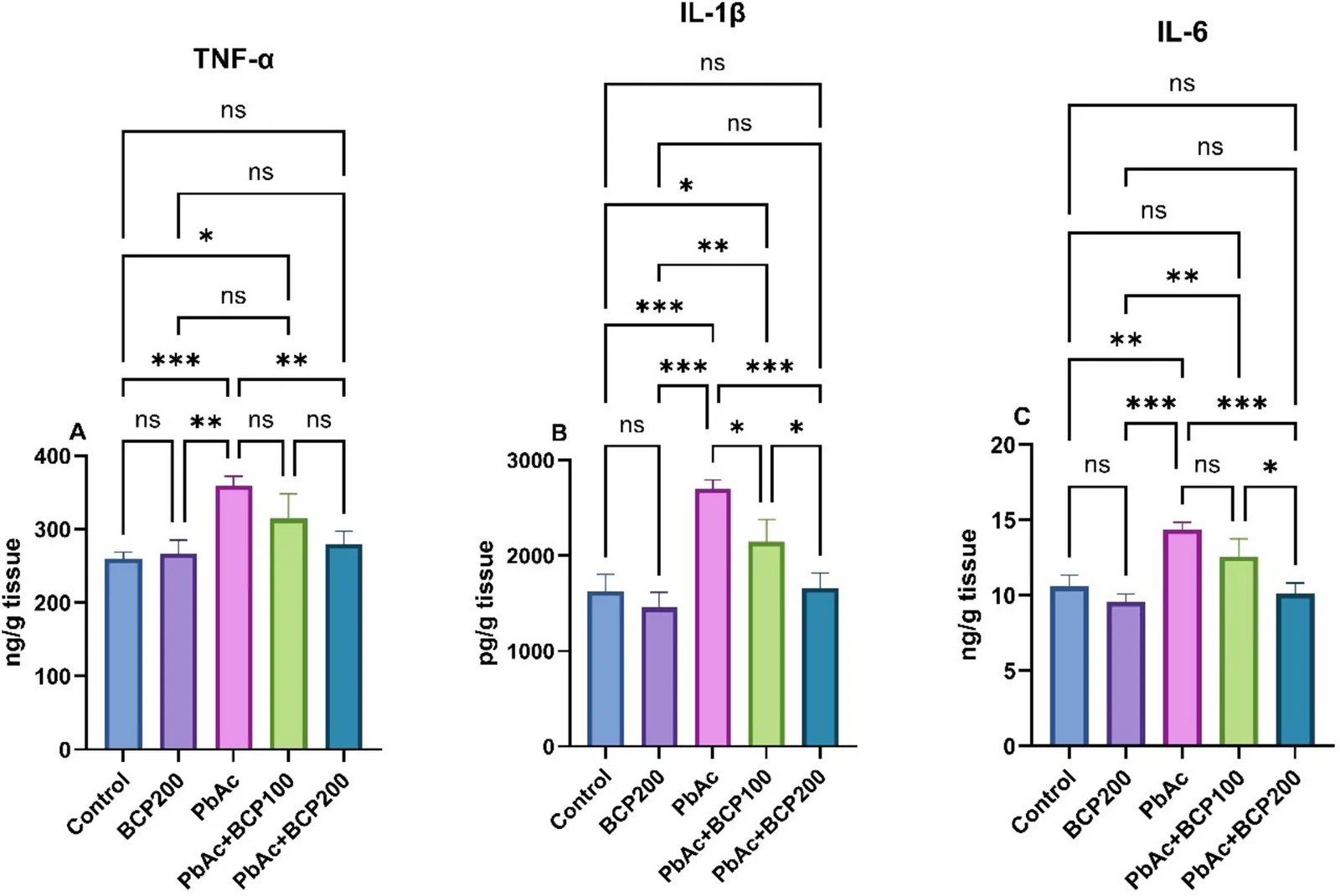
Effects of β-caryophyllene on inflammatory cytokines. Levels of TNF-α, IL-1β, and IL-6 in liver tissues of experimental groups measured by ELISA. Data are expressed as mean ± SD (n = 7). Statistical significance was determined using one-way ANOVA followed by Tukey’s post hoc test. *p < 0.05, **p < 0.01, ***p < 0.001

Serum inflammatory cytokine levels were significantly altered following PbAc administration (Fig. 7). The PbAc-treated group exhibited a significant increase in TNF-α, IL-1β, and IL-6 levels compared with the control group (p < 0.05). Administration of β-caryophyllene significantly attenuated these elevations. Both PbAc + BCP100 and PbAc + BCP200 groups showed significantly lower TNF-α, IL-1β, and IL-6 levels compared with the PbAc group (p < 0.05). Among the treatment groups, the PbAc + BCP200 group demonstrated a more pronounced reduction in inflammatory cytokine levels. No significant differences were observed between the control and BCP200 groups.

### Effects of β-caryophyllene on PI3K/AKT/mTOR signaling and apoptosis-related gene expression in PbAc-induced hepatotoxicity

**Fig. 8:**
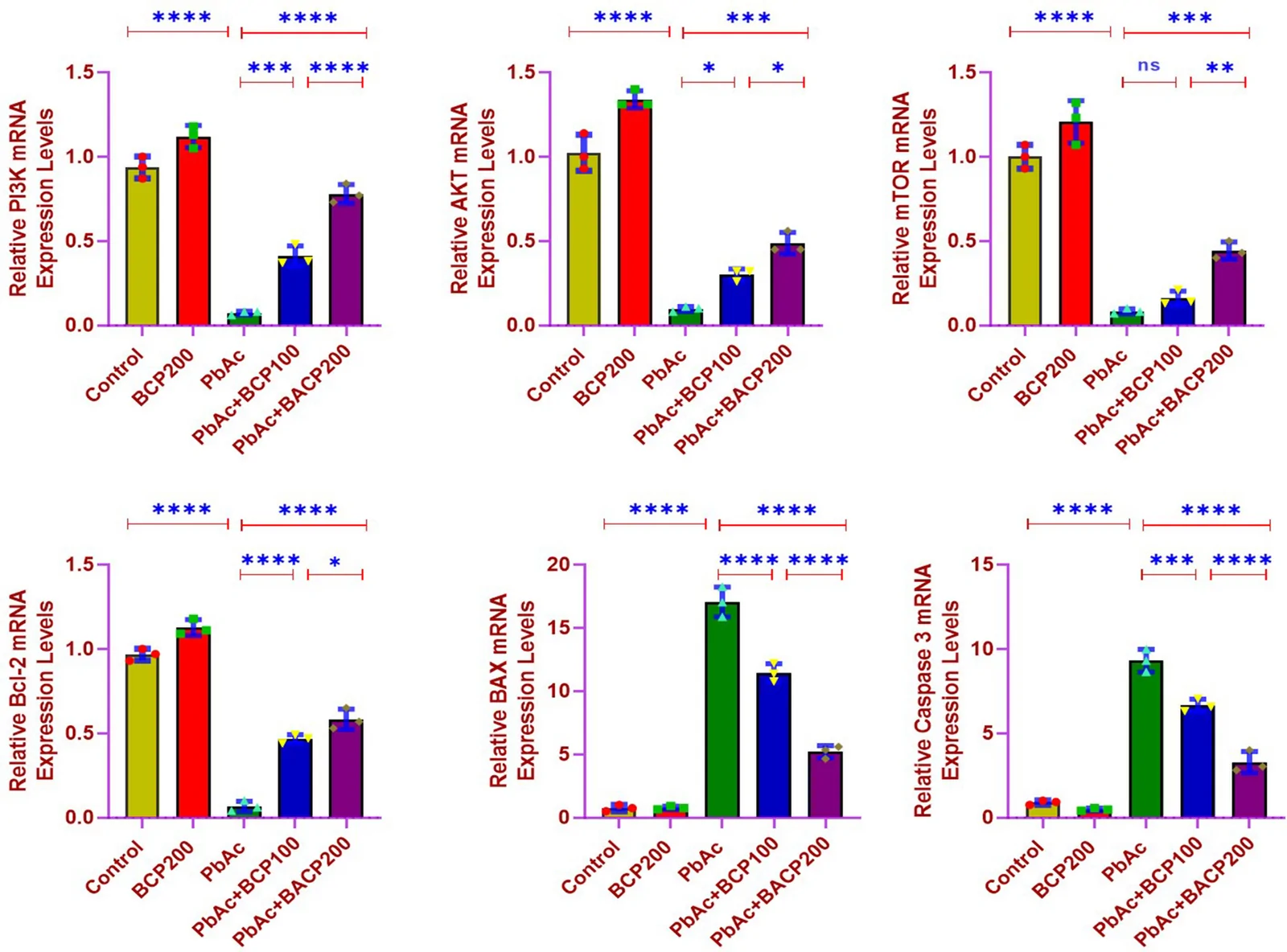
Effects of β-caryophyllene on PI3K/AKT/mTOR signaling and apoptosis-related gene expression. Relative mRNA expression levels of PI3K, AKT, mTOR, Bcl-2, Bax, and Caspase-3 determined by RT-qPCR. Data are presented as mean ± SD (n = 7). Statistical analysis was performed using one-way ANOVA followed by Tukey’s post hoc test. *p < 0.05, **p < 0.01, ***p < 0.001, ****p < 0.0001

Relative mRNA expression levels of PI3K, AKT, and mTOR were significantly altered following PbAc administration (Fig. 8). The PbAc-treated group showed a marked decrease in PI3K, AKT, and mTOR expression levels compared with the control group (p < 0.05). Treatment with β-caryophyllene significantly restored these expression levels. Both PbAc + BCP100 and PbAc + BCP200 groups exhibited significantly higher PI3K, AKT, and mTOR mRNA levels compared with the PbAc group, with the PbAc + BCP200 group showing a more pronounced recovery (p < 0.05).

Similarly, apoptosis-related genes were significantly affected by PbAc exposure. The PbAc group showed a marked increase in Bax and Caspase-3 expression, while Bcl-2 expression was significantly decreased compared with the control group (p < 0.05). Administration of β-caryophyllene significantly reversed these changes. In the PbAc + BCP-treated groups, Bax and Caspase-3 expression levels were significantly reduced, whereas Bcl-2 expression was significantly increased compared with the PbAc group (p < 0.05).

### Histopathological evaluation of liver tissue

**Fig. 9:**
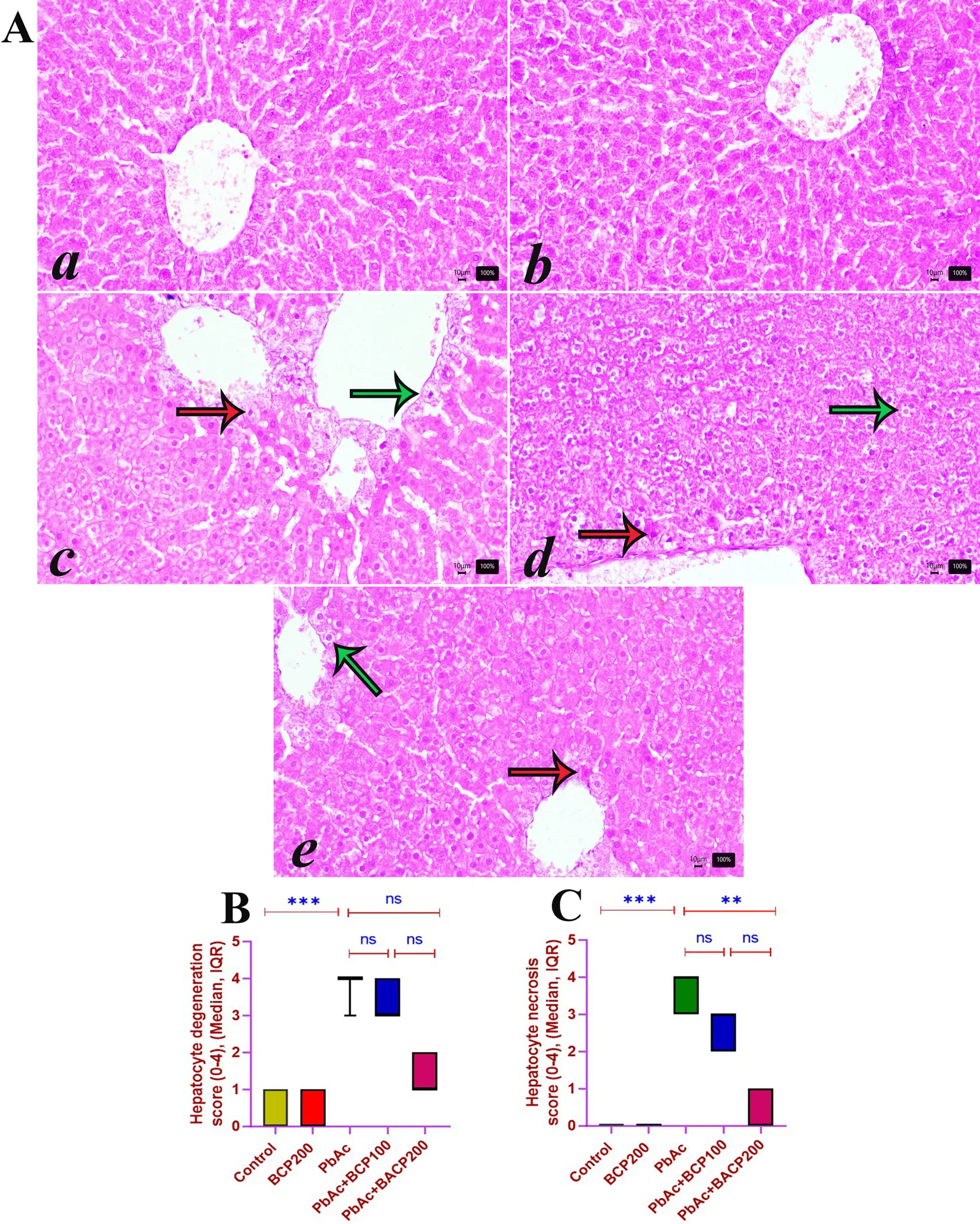
Histopathological findings in liver tissue. Representative H&E-stained liver sections from experimental groups. **(A):** (a) Control group showing normal hepatic architecture. (b) BCP200 group showing normal liver structure similar to control. (c) PbAc group showing hepatocyte degeneration and necrosis around the central vein. (d) PbAc + BCP100 group showing partial improvement in hepatic structure. (e) PbAc + BCP200 group showing marked recovery of hepatic architecture. **(B–C)**: Semi-quantitative scoring of hepatocyte degeneration and necrosis (median, IQR). Statistical analysis was performed using the Kruskal–Wallis test followed by Dunn’s multiple comparison test. *p < 0.05, **p < 0.01, ***p < 0.001; ns: not significant

Histopathological examination of liver tissues revealed normal hepatic architecture in the control and BCP200 groups. Hepatocytes were regularly arranged around the central vein, and no obvious pathological alterations were observed (Fig. 9a–b). In contrast, the PbAc-treated group showed marked histopathological alterations characterized by hepatocyte degeneration and necrosis, particularly around the central vein region (Fig. 9c). Disorganization of hepatic cords and cytoplasmic degeneration were also evident. Treatment with β-caryophyllene attenuated these pathological changes. In the PbAc + BCP100 group, hepatocyte degeneration and necrotic areas were reduced compared with the PbAc group (Fig. 9d). A more pronounced improvement in hepatic morphology was observed in the PbAc + BCP200 group, where hepatic architecture appeared closer to the normal structure (Fig. 9e). Semi-quantitative histopathological scoring supported these observations. The PbAc group exhibited significantly higher hepatocyte degeneration and necrosis scores compared with the control group (p < 0.05). Administration of β-caryophyllene significantly reduced these scores, particularly in the PbAc + BCP200 group (Fig. 9B–C).

The overall findings of this study indicate that β-caryophyllene mitigates PbAc-induced hepatotoxicity by suppressing oxidative stress and inflammation, restoring PI3K/AKT/mTOR signaling, regulating apoptosis, and preserving hepatic histoarchitecture.

## Discussion

The liver is the primary organ in mammals that plays a fundamental role in the detoxification process and, due to this characteristic, is highly sensitive to damage caused by toxic substances. Numerous studies in the literature have shown that lead exposure can cause significant structural and functional abnormalities in liver tissue (Abdallah et al. 2010; Liu et al. 2013). Previous studies have reported that serious hepatic damage develops, particularly in experimental models exposed to lead acetate (Mandal et al. 2023). Biochemical evaluations conducted in the hepatotoxicity model created with lead acetate analyzed ALT, AST, ALP, and GGT levels. A significant increase in ALT and AST levels was observed in the group that developed hepatotoxicity. This situation can be explained by the release of intracellular enzymes into the circulation due to the disruption of hepatocellular membrane integrity. While an increase in ALT is considered an important indicator of hepatocellular damage, an increase in AST levels suggests mitochondrial involvement in addition to hepatocellular damage. An increase in ALP and GGT levels indicates that hepatobiliary functions may also be affected in addition to hepatocellular damage. These biochemical changes are consistent with literature data indicating that lead exposure impairs liver function through oxidative stress and associated cellular damage (Luo et al. 2019). In contrast, the significant decrease in ALT, AST, ALP, and GGT levels in the β-caryophyllene-treated group compared to the PbAc-treated group supports the protective effect of BCP on the liver. This improvement in enzyme levels may be related to the preservation of hepatocellular membrane integrity, reduced oxidative stress, and suppression of the inflammatory process. Similarly, the improvement in serum liver enzymes is consistent with studies reporting that BCP exhibits a functional hepatoprotective effect against lead-induced hepatic damage (Ahmad et al. 2024). The comparatively weaker effects observed at the 100 mg/kg dose of β-caryophyllene may be attributed to a dose-dependent response. It has been reported that the pharmacological activity of β-caryophyllene, particularly its antioxidant and anti-inflammatory effects, increases with higher doses, likely due to improved bioavailability and more effective modulation of cellular signaling pathways. Consistent with our findings, previous studies have demonstrated that higher doses of β-caryophyllene produce more pronounced protective effects in experimental models of oxidative stress and organ toxicity (Espinosa-Ahedo et al. 2022; Bolat et al. 2025a, b, c, d, e, f, g).

One of the most important mechanisms in the development of hepatic damage due to lead exposure is the development of oxidative stress. Lead can cause lipid peroxidation, protein oxidation, and DNA damage by increasing the production of intracellular reactive oxygen species (ROS) (Sannadi et al. 2013; Omobowale et al. 2014). This process can contribute to cell death and the development of an inflammatory response by causing structural and functional abnormalities in hepatocytes. Indeed, various studies have shown that lead exposure triggers cellular damage by increasing ROS production (Liu et al. 2025; Tekin et al. 2025). BCP has been reported to exhibit a significant antioxidant effect in cases of hepatic oxidative stress associated with heavy metal exposure (Elgharib et al. 2025; Bolat et al. 2025f). The decrease in lipid peroxidation and the increase in antioxidant defense systems such as SOD and GSH indicate that this compound supports the oxidant-antioxidant balance (Bolat et al. 2025f). This effect is thought to be related to the activation of the Nrf2/HO-1 signaling pathway and the consequent increase in antioxidant gene expression. Our study determined that lead exposure increases MDA levels, indicating the formation of oxidative stress. In contrast, BCP administration was found to suppress this increase, thereby reducing oxidative stress levels. These findings support the protective potential of BCP against lead-induced liver damage. In addition, Sirt1 has been reported to play a regulatory role in the activation of the Nrf2/HO-1 signaling pathway. Sirt1 can enhance Nrf2 activity by promoting its nuclear translocation and stability, thereby facilitating the transcription of antioxidant response genes such as HO-1. In this context, the decrease in Sirt1 expression observed in the PbAc-treated group may contribute to the suppression of Nrf2/HO-1 signaling and the weakening of cellular antioxidant defense. Conversely, the restoration of Sirt1 expression following β-caryophyllene treatment may support the activation of the Nrf2/HO-1 pathway, thereby contributing to the reduction of oxidative stress and the enhancement of hepatocellular protection. These findings suggest a coordinated interaction between Sirt1 and the Nrf2/HO-1 axis in mediating the protective effects of β-caryophyllene (Yang et al. 2016; Chen et al. 2023).

In our study, we also evaluated oxidative DNA damage and the expression of cellular redox regulators 8-OHdG and Keap1. Previous studies have reported increased 8-OHdG expression in liver tissue damaged by lead acetate, leading to oxidative DNA damage (Pawlas et al. 2017). In addition, it has been suggested that the increase in Keap1 expression is associated with the suppression of antioxidant defense mechanisms and the disruption of cellular redox balance (Jiang et al. 2021). In the present study, while lead exposure was observed to increase 8-OHdG and Keap1 expression, BCP administration was found to suppress this increase. These findings suggest that BCP may exert a protective effect in hepatocytes by reducing oxidative stress and DNA damage.

This study also observed that the Nrf2/HO-1 signaling pathway, one of the key regulators of antioxidant defense, was suppressed by PbAc exposure. The literature also reports that lead increases ROS production, causing an imbalance between Nrf2 and Keap1 in favor of Keap1, which weakens antioxidant defense mechanisms (Omobowale et al. 2014; Long et al. 2016). In contrast, the BCP application was observed to increase Nrf2 and HO-1 expression again. This suggests that BCP strengthens the antioxidant defense system by reducing oxidative stress and rebalancing Keap1/Nrf2. Similarly, several studies have reported that BCP enhances the antioxidant response by activating the Nrf2 signaling pathway (Da Silveira et al. 2021; Bolat et al. 2025c).

Previous studies have demonstrated that β-caryophyllene has inhibitory effects on the inflammatory response (Matacchione et al. 2021). In particular, the reduction of oxidative stress through the activation of the Nrf2/HO-1 pathway may contribute to the suppression of inflammatory processes (Baradaran Rahimi and Askari 2022; Baradaran Rahimi et al. 2023; Jiang et al. 2024; Kamel et al. 2024). In our study, the significant increase in TNF-α, IL-1β, and IL-6 levels in the groups treated with lead acetate indicates that lead exposure triggers an inflammatory response in liver tissue. It has been reported that dysregulation in the Keap1/Nrf2/HO-1 axis, along with increased oxidative stress, may contribute to the intensification of the inflammatory response (Moghadam Aghajari and Asle-Rousta 2025). İn contrast of, the decrease observed in TNF-α, IL-1β, and IL-6 levels in groups treated with BCP supports the anti-inflammatory effect of BCP. This effect is thought to be related to BCP's ability to enhance antioxidant defense and thus suppress inflammatory cytokine production.

Significant changes in the expression levels of the PI3K/AKT/mTOR signaling pathway were observed in the hepatotoxicity model created with lead acetate. The suppression observed in PI3K, AKT, and mTOR expression in the group that developed hepatotoxicity suggests that lead exposure negatively affects cellular growth, metabolic balance, and survival mechanisms in hepatocytes (Tewari et al. 2022). It has been reported that oxidative stress can suppress this signaling pathway by disrupting the cellular redox balance, which may contribute to the progression of damage in hepatocytes (Tekin et al. 2026). In contrast, the fact that PI3K, AKT, and mTOR expression levels in the BCP-treated group approached those of the control group suggests that BCP may enhance the adaptation of hepatocytes to stress conditions by supporting cellular protective signaling pathways.

**Fig. 10:**
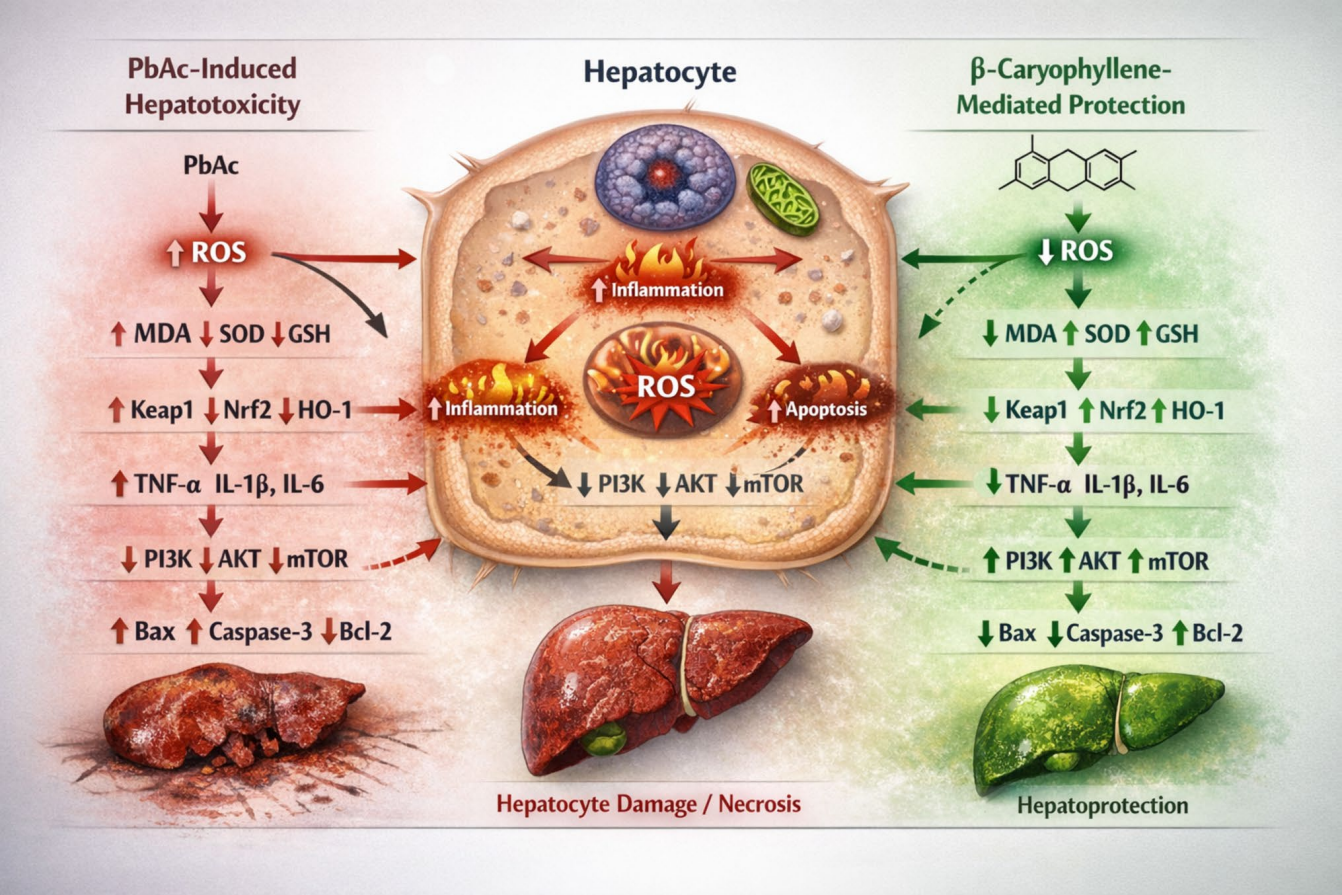
Schematic illustration of the proposed molecular mechanisms underlying β-caryophyllene-mediated protection against PbAc-induced hepatotoxicity

When apoptosis markers were evaluated, it was determined that Bax and Caspase-3 expression increased and antiapoptotic Bcl-2 levels decreased in the group treated with lead acetate. These findings indicate that lead exposure triggers apoptotic cell death in hepatocytes (Tekin et al. 2025). Previous studies have also reported that oxidative stress and mitochondrial dysfunction activate apoptotic mechanisms. In contrast, increased Bcl-2 expression and decreased Bax and Caspase-3 levels in the BCP-treated group suggest that BCP may limit cellular loss in hepatocytes by suppressing the apoptotic process (Bolat et al. 2025a, b, c, d, e, f, g; Moghadam Aghajari and Asle-Rousta 2025). Collectively, these findings suggest that β-caryophyllene mitigates PbAc-induced hepatotoxicity through a multi-target mechanism involving suppression of oxidative stress and inflammation, restoration of Nrf2/HO-1 and PI3K/AKT/mTOR signaling pathways, and regulation of apoptosis. The integrated molecular interactions underlying these protective effects are summarized schematically in Fig. 10.

## Conclusion

The present findings suggest that β-caryophyllene may exert significant hepatoprotective effects against lead-induced hepatotoxicity. This protective effect may be related to the suppression of oxidative stress, the enhancement of Nrf2/HO-1-mediated antioxidant defense mechanisms, the support of the PI3K/AKT/mTOR signaling pathway associated with cellular survival, and the balancing of apoptotic processes. Through these mechanisms, β-caryophyllene may contribute to the preservation of hepatocellular integrity and maintenance of liver function under lead-induced oxidative injury conditions. Overall, the findings support the potential protective role of β-caryophyllene in heavy metal-induced liver injury; however, further mechanistic and protein-level studies are required to confirm these effects.

Despite these promising findings, this study has some limitations. The molecular mechanisms were primarily evaluated at the gene expression level, and protein-level confirmation (e.g., Western blot analysis) was not performed. In addition, the experimental model was limited to an acute hepatotoxicity design, and long-term effects were not investigated. Future studies should focus on validating these findings at the protein level, exploring dose-dependent responses in more detail, and investigating the long-term hepatoprotective effects of β-caryophyllene in chronic exposure models.

## Data Availability

All source data for this work (or generated in this study) are available upon reasonable request.
